# Hantaviral Proteins: Structure, Functions, and Role in Hantavirus Infection

**DOI:** 10.3389/fmicb.2015.01326

**Published:** 2015-11-27

**Authors:** Musalwa Muyangwa, Ekaterina V. Martynova, Svetlana F. Khaiboullina, Sergey P. Morzunov, Albert A. Rizvanov

**Affiliations:** ^1^Institute of Fundamental Medicine and Biology, Kazan Federal UniversityKazan, Russia; ^2^Nevada Center for Biomedical Research, RenoNV, USA; ^3^Department of Pathology and Nevada State Public Health Laboratory, University of Nevada School of Medicine, RenoNV, USA

**Keywords:** Hantavirus, nucleocapsid protein, glycoprotein, reassortment, MxA protein

## Abstract

Hantaviruses are the members of the family Bunyaviridae that are naturally maintained in the populations of small mammals, mostly rodents. Most of these viruses can easily infect humans through contact with aerosols or dust generated by contaminated animal waste products. Depending on the particular Hantavirus involved, human infection could result in either hemorrhagic fever with renal syndrome or in Hantavirus cardiopulmonary syndrome. In the past few years, clinical cases of the Hantavirus caused diseases have been on the rise. Understanding structure of the Hantavirus genome and the functions of the key viral proteins are critical for the therapeutic agents’ research. This paper gives a brief overview of the current knowledge on the structure and properties of the Hantavirus nucleoprotein and the glycoproteins.

## Introduction

Hantaviruses comprise genus *Hantavirus* within family Bunyaviridae (Elliott, 1990). Humans become infected by either inhaling virus contaminated aerosols or having contact with the urine or droppings of infected animals (Jonsson et al., 2010). In humans hantaviruses cause either hemorrhagic fever with renal syndrome (HFRS) or Hantavirus cardiopulmonary syndrome (HCPS). Generally, each distinct Hantavirus is maintained in nature in the populations of the particular small mammal (rodent or insectivore) host species. Murinae-associated hantaviruses cause HFRS, while Sigmodontinae associated hantaviruses usually cause HCPS. Most of the Arvicolinae-borne hantaviruses (Prospect Hill virus and Tula virus being the most prominent ones) seem to be non-pathogenic for humans (Plyusnin et al., 1994; Schmaljohn and Hjelle, 1997). In accord with the geographic distribution of the virus specific natural hosts, HFRS is mainly diagnosed in Europe and Asia, with murine-borne Hantaan virus (HTNV), Dobrava-Belgrade virus (DOBV), and Seoul virus, as well as arvicoline-borne Puumala virus (PUUV), serving as the main causative agents. HCPS is endemic in the Americas and is caused by a variety of the Sigmodontinae-borne New World Hantaviruses, with Andes virus (ANDV) and Sin Nombre virus (SNV) being the most prominent sources of human infections. Mortality rates vary from 0.3 to 10% for HFRS and between 30 and 40% for HCPS (Jonsson et al., 2010; Macneil et al., 2011; Krautkramer et al., 2013). HFRS clinical symptoms include fever, renal dysfunction, haemorrhagic manifestations, and shock. HCPS is characterized by fever, myalgia, headache, and gastrointestinal symptoms, followed by non-cardiogenic pulmonary oedema, and shock. A summary of the geographic distribution and host affiliation of the most prominent hantaviruses and the diseases they cause is given in **Table 1**.

**Table 1:** Representative hantaviruses and their rodent hosts.

Rodent host subfamily	Rodent host species	Virus species	Disease	Geographic distribution
Murinae	*Apodemus agrarius*	Hantaan	HFRS	Russia, China, and Korea
Murinae	*A. flavicollis*	Dobrava-Belgrade	HFRS	Balkans and Europe
Murinae	*Rattus norvegicus*	Seoul	HFRS	Worldwide
Sigmodontinae	*Peromyscus maniculatus*	Sin Nombre	HCPS	Western Canada and USA
Sigmodontinae	*P. maniculatus*	Monongahela	HCPS	Eastern Canada and USA
Sigmodontinae	*P. leucopus*	New York	HCPS	USA
Sigmodontinae	*P. leucopus*	Blue River	HCPS	USA
Sigmodontinae	*Sigmodon hispidus*	Black Creek Canal	HCPS	USA
Sigmodontinae	*Oryzomys palustris*	Bayou	HCPS	USA
Sigmodontinae	*S. hispidus*	Muleshoe	HCPS	USA
Sigmodontinae	*S. alstoni*	Cano Delgadito	Not known	Venezuela
Sigmodontinae	*Oligoryzomys longicaudatus*	Andes	HCPS	Argentina, Chile, and Uruguay
Sigmodontinae	*O. longicaudatus*	Oran	HCPS	Argentina
Sigmodontinae	*O. Flavescens*	Lechiguanas	HCPS	Argentina and Uruguay
Sigmodontinae	*Calomys laucha*	Laguna Negra	HCPS	Paraguay and Bolivia
Arvicolinae	*Clethrionomys glareolus*	Puumala	HFRS	Russia, Europe, and Asia
Arvicolinae	*Microtus pennsylvanicus*	Prospect Hill	Not known	North America
Arvicolinae	*M. ochrogaster*	Blood Land Lake	Not known	North America
Arvicolinae	*M. arvalis*	Tula	Not known	Europe, Russia, and Asia
Arvicolinae	*M. californicus*	Isla Vista	Not known	USA and Mexico


## Hantavirus Genome Structure And Life Cycle

Hantavirus virions have spherical shape with size varying between 80 and 120 nm. Hantavirus genome is comprised of three segments of single stranded negative sense RNA. Based on their size, these three segments are named small (S), medium (M), and large (L). L segment encodes viral polymerase, while M and S segments encode the precursor (GPC) for two viral surface glycoproteins (G1 and G2, or alternatively called Gn and Gc), and the nucleocapsid (N) protein, respectively. Each virion generally contains equimolar amounts of genomic RNA, with a single molecule of the viral RNA-dependent RNA polymerase (RdRp) being attached to each segment of viral RNA. All viral RNA segments are coated with the molecules of the N protein forming ribonucleoproteins (RNPs; Elliott, 1990). These are enclosed by an envelope consisting of a lipid bilayer, with G1 and G2 surface glycoproteins embedded into it (Elliott, 1990).

**FIGURE 1:**
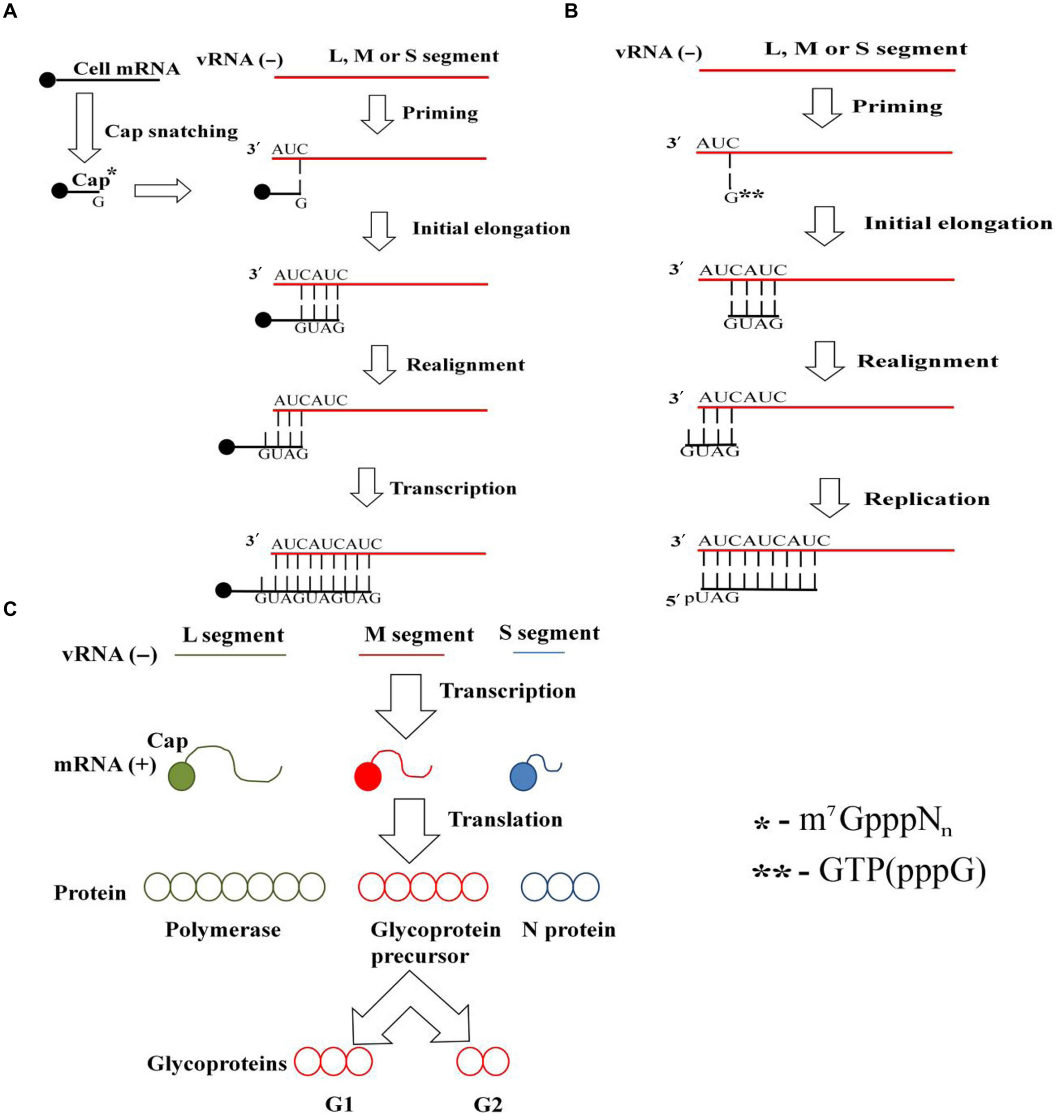
**Hantavirus transcription, replication and translation.** **(A)** Hantavirus transcription. Transcription occurs through a prime and realign mechanism. Cellular mRNA is cleaved by either Hantavirus RNA-dependent RNA polymerase (RdRp) or cellular endonucleases in a process called cap snatching, thus forming a capped primer (m^7^GpppN_n_). It is this capped primer that initiates transcription by aligning its guanidine to the 3′ cytosine of the vRNA. After synthesis of several nucleotides, the nascent RNA slips back and realigns. Final elongation then takes place, producing an extra copy of viral mRNA. **(B)** Replication of Hantavirus RNA. Replication takes place in cytoplasm of the infected cell, using prime and realign mechanism. RdRp attached to the 3′ end of vRNA aligns guanidine triphosphate (pppG) residue to the first cytosine of the virus RNA and synthesizes the first three nucleotides of the new cRNA strand. The nascent RNA slips back and realigns after successive addition of bases. Then, final elongation takes place, resulting in production of the full length cRNA. In turn, this positive strand anti-genomic cRNA serves as a template for producing a large amount of the new strands of vRNA. **(C)** Hantavirus transcription and translation. Negative sense viral RNA serves as a template for the viral RdRp, which initiates transcription by cap-snatching mechanism and generates viral mRNA. Viral mRNAs are translated producing N protein, glycoprotein precursor (which is cleaved to form G1 and G2 glycoproteins), and RdRp from the small (S), medium (M), and large (L) segment-originated mRNA, respectively.

**FIGURE 2:**
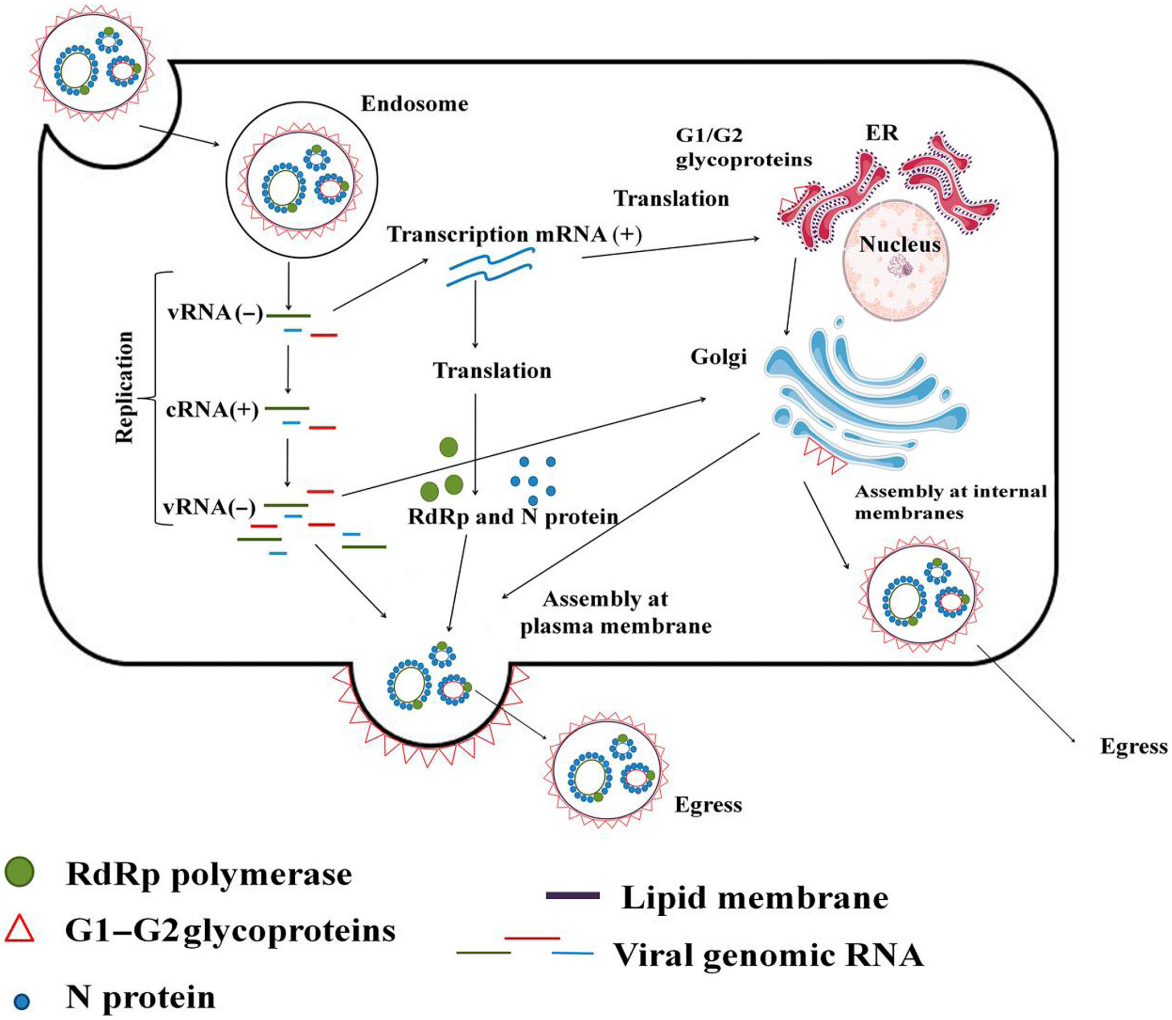
**Hantavirus life cycle.** Virion binds to the cell surface membrane receptor and enters the cell via endocytosis. Once inside the cell, RNPs are released from the late endosome via pH-mediated membrane fusion. Virion-supplied RdRp is driving initial mRNA transcription which takes place in cytoplasm. Viral genomic (minus sense) RNA serves as a template for generation of mRNA utilized for protein synthesis. When sufficient amounts of the viral proteins are produced, RdRp switches to the replication mode synthesizing full length anti-genomic (plus sense) RNA, which in turn serves as a template for producing a large amount of the new full length minus sense viral RNA molecules. Newly synthesized vRNA becomes encapsidated with N protein forming ribonucleoprotein and transported into perinuclear membrane system, from where they will be transported to Golgi for initiation of virion formation. Egress takes place at the plasma membrane.

Hantavirus virion attachment to the host cell via cellular receptors is followed by endocytosis. RNPs are released to the cytoplasm from the late endosome following pH-mediated membrane fusion. Transcription and translation take place either at the site of RNPs release or at the endoplasmic reticulum–Golgi intermediate compartment (ERGIC). In case of the latter, the RNPs are transported to ERGIC. The viral polymerase, RdRp, possesses transcriptase, replicase and endonuclease functions; thus, it carries out both virus transcription (**Figure 1A**) and replication (**Figure 1B**). To initiate transcription, RdRp cleaves cellular mRNA forming capped primers. Recently, cellular endonucleases have also been suggested to participate in capped primer formation by cleaving cellular mRNAs which cap-structures are protected from degradation by the specifically bound viral N protein (Mir et al., 2008). These capped primers initiate transcription of viral mRNAs. S segment derived mRNA serves as a template for the N protein, and for some particular hantaviruses also it produces a non-structural NSs protein (**Figure 1C**). M segment derived mRNA produces GPC on the ER membrane-bound ribosomes. G1 and G2 glycoproteins are transported from the ER to the Golgi complex or to the plasma membrane where assembly takes place (**Figure 2**). Old World hantaviruses assemble at the Golgi while New World hantaviruses assemble at the plasma membrane (Elliott, 1990; Ravkov et al., 1998; Spiropoulou, 2013) (**Figure 2**). After assembly, the newly formed envelope contains spike-like projections (Elliott, 1990; Welsch et al., 2007; Hepojoki et al., 2012) formed by the tetramers of the viral surface glycoproteins, which apparently play an important role in both virus assembly and cell entry (Welsch et al., 2007; Lyles, 2013; Yamauchi and Helenius, 2013). Newly assembled virions are released through exocytosis.

## Structure And Properties Of Hantavirus Glycoproteins

Hantavirus surface glycoproteins G1 and G2 are coded by the M segment and are expressed as a polyprotein precursor, GPC, which is cleaved by a cellular protease during translocation to ER yielding mature G1 and G2 glycoproteins (**Figure 1C**) (Pensiero and Hay, 1992; Plyusnin et al., 1996). Cryo-electron microscopy and cryo-electron tomography studies have shown that G1 and G2 proteins form square-shaped surface spikes protruding from the viral membrane, with each spike complex made of four G1 and four G2 subunits (Huiskonen et al., 2010; Battisti et al., 2011). It has been shown that these G1/G2 glycoprotein heterodimers may interact with specific cellular surface proteins, β_3_-integrins, facilitating cellular entry of HCPS-causing hantaviruses (Gavrilovskaya et al., 1998).

Both G1 and G2 glycoproteins are built in a similar way, each containing a large globular domain, a hydrophobic transmembrane sequence and a small C-terminal cytoplasmic tail. Since no matrix proteins exist connecting Hantavirus nucleoproteins and envelope proteins, it is suggested that there is a direct interaction between N protein and cytoplasmic tails of G1 and G2. Nuclear magnetic resonance spectroscopy has shown that a part of the G1 tail (residues 543–599) has a double ββα-fold zinc finger made up of a highly conserved motif that has high similarity between ANDV and Prospect Hill viruses (PHV) (Estrada et al., 2009, 2011). It has been suggested that these zinc fingers play a role in virus assembly (Estrada and De Guzman, 2011).

## Glycoprotein Interaction And Trafficking

Maturation of Bunyavirus glycoproteins takes place in the Golgi complex (Pettersson and Melin, 1996). During maturation, G1 and G2 glycoproteins are N-glycosylated. Three glycosylation sites are located on G1 and only one on G2 glycoprotein. Both G1 and G2 glycoproteins are sensitive to endoglycosidases H and F. It has been reported that G1 and G2 targeting to Golgi depends on conformational interaction between these two glycoproteins (Shi and Elliott, 2002; Deyde et al., 2005). Furthermore, it appears that G1 glycoprotein plays an important role in facilitating Golgi trafficking of both glycoproteins. For example, when G1 glycoprotein is expressed individually, it becomes partially localized to Golgi, while the majority of this protein is localized in the endoplasmic reticulum (Deyde et al., 2005). However, when G2 is individually expressed, it becomes exclusively localized in the endoplasmic reticulum (Ruusala et al., 1992; Spiropoulou et al., 2003). It is important to note that glycoproteins from different hantaviruses are capable to interact resulting in proper targeting to the Golgi complex (Deyde et al., 2005).

## Glycoprotein-Induced Virulence

Viral infection ignites innate immune responses aimed to reduce viral replication. Type I interferons (IFNs) play a pivotal role in providing direct antiviral protection as well as activating natural killer (NK) cells, the key effector cells of the innate immune response. On the other hand, in order to survive viruses develop mechanisms to prevent elimination by inhibiting pathways activating type I IFN transcription (Finlay and McFadden, 2006; Yoneyama and Fujita, 2007). It has been shown that expression of the G1 protein cytoplasmic tail of pathogenic hantaviruses (Alff et al., 2006) inhibits the induction of IFN-β. This ability distinguishes pathogenic hantaviruses from non-pathogenic ones, as the latter are incapable of inhibiting IFN-β induction (Alff et al., 2006). The G1 cytoplasmic tail of the pathogenic hantaviruses has been shown to inhibit IFN-β transcription by binding to TRAF3 (Alff et al., 2008) and preventing RIG-I/TBK1-directed IRF3 phosphorylation (Mackow et al., 2014; Matthys et al., 2014). TRAF3 is an E3 ubiquitin ligase that forms a TBK1–TRAF3 complex, which is crucial for IRF3 phosphorylation. Phosphorylation of IRF3 is vital in IFNβ induction.

## Hantavirus Reassortment And Compatibility Of The Hantavirus Proteins

Reassortment, i.e., exchange of the genome segments between different virus strains, plays an essential role in maintaining segmented viruses and can produce new strains with novel characteristics and improved survivability. The evolution of the Rift Valley Fever virus (RVFV) presents an example of how novel reassortants can be naturally generated in endemic areas (Sall et al., 1997). Rapid virus evolution caused by such reassortment may bring about global outbreaks (Webster and Laver, 1971; Young and Palese, 1979). Also, ability to generate reassortants may put people living in the endemic areas at the risk of generating uncontrolled chimeric viruses by using live attenuated vaccines (Sall et al., 1997). Initially, genetic reassortment has been shown among members of arthropod-borne Bunyaviridae (Beaty et al., 1985; Chandler et al., 1991). Later on, genetic reassortment between different Hantavirus strains in nature has been documented as well (Henderson et al., 1995; Li et al., 1995). Li et al. (1995) proposed that such reassortment could lead to the emergence of new Hantavirus strains with novel epidemiological characteristics.

Reassortment is most likely to occur between genetically different strains of the same Hantavirus, or between closely related hantaviruses circulating within closely related rodent host species. Despite being rather rare, reassortment among hantaviruses that are distantly related is also possible in nature. It is well-known that Hantavirus infection could “spill over” to the non-specific host when different animal species share the same ecological niche. This could potentially result in dual infection of a single animal. Replication of two different hantaviruses in the same host organism may produce reassortants with new characteristics (Henderson et al., 1995; Rodriguez et al., 1998).

**FIGURE 3:**
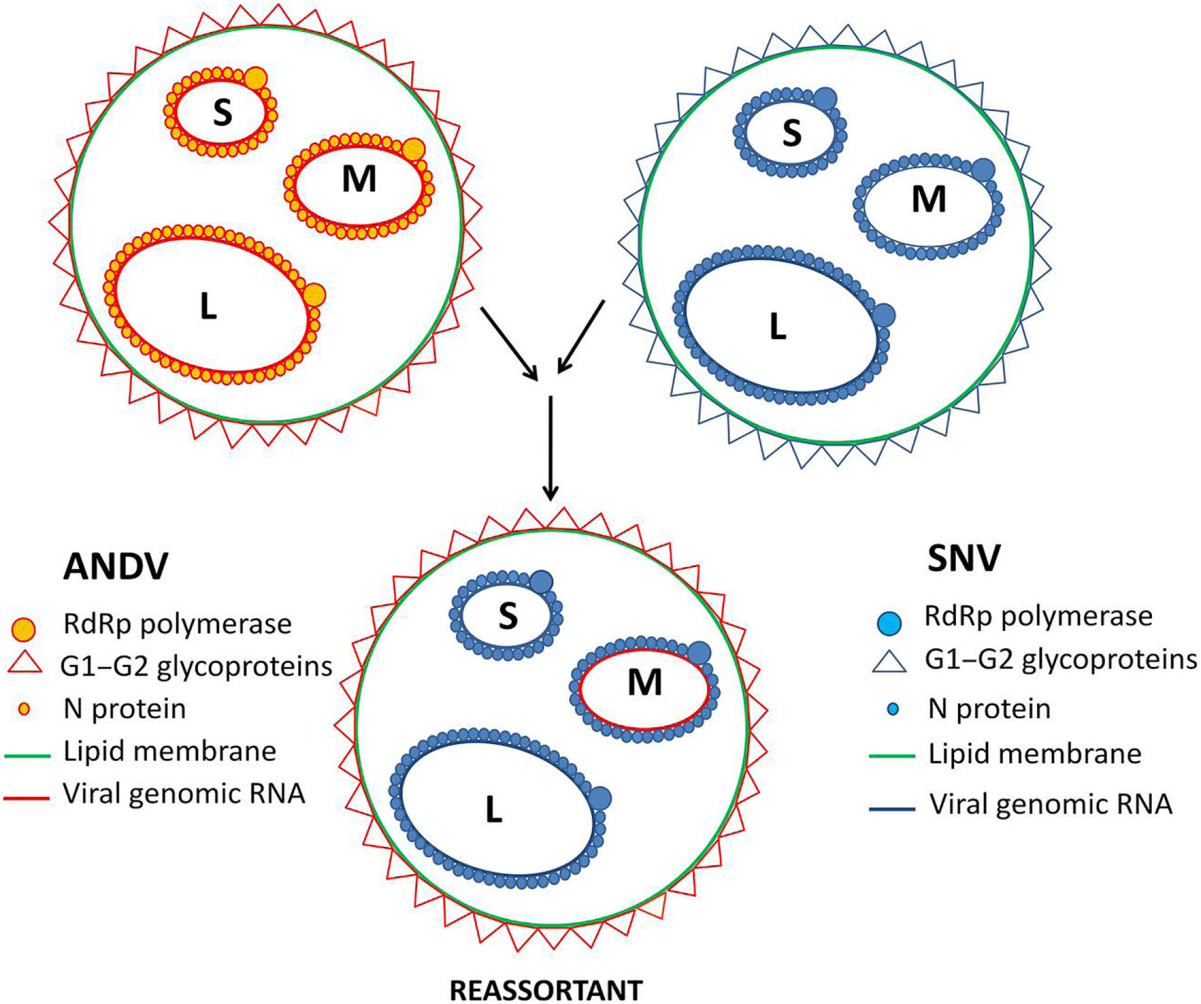
**Hantavirus reassortment.** Infection of one host with two different Hantavirus strains may result in reassortment. Reassortment has been shown between ANDV and SNV resulting in reassortants possessing ANDV M segment and SNV L and S segment.

*In vitro* studies have proven that genetic reassortants can be developed between distantly related hantaviruses (Rodriguez et al., 1998; Kang et al., 2002). In details, the ability of two distantly related hantaviruses to develop reassortants *in vitro* have been investigated by Rizvanov et al. (2004). The authors have shown that ANDV and SNV (**Figure 3**) can generate reassortants with novel infectivity characteristics which differ from both parental strains. Noteworthy, the resulting reassortant viruses always retained the S and L segments of the same parental Hantavirus, while the M segment was introduced from the other one. In spite of both viruses being *Sigmodontinae*-borne, their rodent hosts are separated geographically. SNV circulates in North America, while ANDV endemic for South America (Plyusnin and Morzunov, 2001). These data are in agreement with the previous finding published by Urquidi and Bishop (1992) demonstrating that the reassortant progeny between Bunyaviruses contains homologous S and L segments. Few progeny contain S and L segments that are heterozygous, i.e., virion contains corresponding segments from both parental strains. Virus progeny of the hantaviruses that are closely related have a different prevalence of homologous L and S segments from those that are distantly related (Rodriguez et al., 1998; Scmaljohn and Hopper, 2001).

The M segment plays an important role in Bunyavirus replication (Beaty et al., 1981) and is also known to alter the efficiency of virus budding. Glycoproteins encoded by the M segment take part in cell surface attachment, thus, they are essential for viral entry into the host cell. It is interesting to note that the stable reassortment progeny between SNV and ANDV contained the M segment from the virus which has higher capacity to replicate in the host cell type used in the experiments (Rizvanov et al., 2004). This data supports the notion that reassortment strategy utilized by segmented viruses may generate novel virus strains with higher capacity to propagate. Two additional conclusions that could be drawn from the reassortment experiments are: (i) the viral RdRp obviously works much better on the viral RNA template coated with the homologous N protein, and (ii) cytoplasmic tails of the Hantavirus G1/G2 glycoproteins seem to interact with the heterologous RNPs at least as efficiently as with the homologous ones.

Ability to generate reassortants between distantly related hantaviruses provides a tool for studying the role of each viral segment (and corresponding viral protein) in pathogenesis of infection. Also, reassortants can be used for analyzing the Hantavirus specificity to the animal host.

## Structure And Properties Of The Hantavirus N Protein

The Hantavirus N protein consists of approximately 433 amino acid residues (about 50 kDa in size). The N protein appears to be highly conserved among different hantaviruses. It has been shown that large amounts of N protein are expressed early after infection (Vapalahti et al., 1995). Also, it has been demonstrated that early immune response in Hantavirus patients is directed mainly against N protein. Therefore, many virus diagnostics developed are based on detecting Hantavirus N protein or anti-N protein antibody (Amada et al., 2013; Yoshimatsu and Arikawa, 2014).

The N protein is expressed exclusively in the cytoplasm (Elliott et al., 2000) of the infected cell. Hantavirus N protein plays a pivotal role in the virus life cycle as it is required for encapsidating viral RNA, as well as regulating virus replication and assembly.

## RNA Binding And Ribonucleoprotein Assembly

The N protein protects viral genomic RNA from degradation by cellular nucleases by forming viral RNPs. The mechanisms of RNA encapsidation are not completely understood. It has been shown that the N protein selectively interacts with Hantavirus RNA, encapsidating vRNA (negative sense genomic) and cRNA (positive sense anti-genomic) while leaving viral mRNA free. Selective encapsidation is thought to be possible due to presence of the unique panhandle terminal structure formed by the self-complimentary terminal sequences of the full length vRNA and cRNA. It has been demonstrated that these 23 nucleotides-long terminal sequences can serve as a binding site for the viral RdRp and have high affinity to the N protein (Mir and Panganiban, 2004). In particular, the N protein of some hantaviruses, such as HTNV, has been proven to preferably bind to its S segment vRNA rather than to the S segment open-reading frame or non-specific RNA. This may suggest that the N protein recognition site resides in the non-coding region of HTNV vRNA (Severson et al., 1999). It was later reported that such binding depends on the 5′ end sequence of the S segment vRNA (Severson et al., 2001).

## N Protein Interacts With Human MxA(p78) Protein

It has been shown that the efficiency of Hantavirus replication is inversely proportional to the ability of infected cells to activate MxA expression (Kanerva et al., 1996). MxA protein is a key component of the type I IFN-induced antiviral state providing resistance to a wide range of the RNA viruses (Pavlovic et al., 1993). There are two types of Mx proteins in humans, MxA and MxB (Horisberger et al., 1988), with only MxA known to possess anti-viral activity (Haller and Kochs, 2002). Interferon regulatory factor 3 (IRF-3) regulates activation of MxA gene transcription (Baigent et al., 2002). Generally, IRF-3 is present in the cytoplasm of the cell in a dormant state (Reich, 2002). However, upon infection, IRF-3 translocates into the nucleus, where it initiates transcription of MxA and other IFN inducible genes (Reich, 2002; Melchjorsen and Paludan, 2003). It has been shown that IRF-3 nuclear translocation can occur as early as 24 after Hantavirus infection (Khaiboullina et al., 2005).

MxA activation has been shown to vary in different cell types (Khaiboullina et al., 2005). For example, high MxA activation level was demonstrated in human umbilical cord endothelial cells (HUVECs), while ativation of MxA in VeroE6 cells was virtually undetectable. Further studies have shown that Hantavirus replication efficacy is inversely proportional to the ability of infected cells to activate expression of MxA protein (Kanerva et al., 1996; Khaiboullina et al., 2005). These data suggest that variations in Hantavirus replication may partially depend on ability of the particular cell types to activate MxA protein. In turn, MxA protein is known to bind to the N protein forming an MxA/N protein complex (Khaiboullina et al., 2005). Formation of MxA/N complexes has been suggested for some other Bunyaviruses as a potential mechanism of MxA inhibition of viral replication (Kochs et al., 2002). Thus, it is very likely that in the case of hantaviruses the mechanism of MxA inhibition is similar.

## Hantavirus Infection Activates The Innate Immune Response

Increased microvascular permeability is characteristic for hantavirus infections (Zhang et al., 1987; Enria et al., 2001; Hepojoki et al., 2014). However, permeability of endothelial cell monolayer did not change after Hantavirus infection *in vitro* (Khaiboullina et al., 2000; Sundstrom et al., 2001). Hantavirus infection is not cytopathic, therefore, it has been suggested that an increased microvascular leakage is most likely associated with cell response to infection, rather than related to virus replication. A DNA microarray conducted to determine changes in cell responses in Hantavirus infected cells showed that non-pathogenic (PHV) and pathogenic (SNV) hantaviruses have different effects on transcriptional activity in infected cells (Khaiboullina et al., 2004). In particular, it has been shown that PHV infection activates approximately five times less genes than the SNV infection does (36 genes were up-regulated in PHV-infected cells in comparison to 175 genes in SNV-infected cells). As infection progressed, more changes in transcriptional activation were detected.

Activation of nuclear and transcriptional factors was shown to vary in cells infected with pathogenic versus non-pathogenic hantaviruses (17 vs. 8; Khaiboullina et al., 2004). Also, Hantavirus infection activates IRF-7, IRF-1, and IRF-9 transcription factors (Khaiboullina et al., 2004). Interestingly, the transcriptional activity of these factors was lower in non-pathogenic (PHV) than in pathogenic (SNV) Hantavirus. Although no changes in transcriptional activity of IRF3 were noted, nuclear translocation of this factor in Hantavirus infected cells has been shown by immunohistochemistry (Khaiboullina et al., 2005). Nuclear translocation is essential for IRF3 activity which includes activation of IFN inducible genes as well as activation of cytokines. It has been demonstrated that IRF-3 controls activation of CCL5 gene transcription, while IRF-1 and IRF-3 regulate expression of MxA protein (Baigent et al., 2002). Up-regulation of CCL5 and MxA has been shown in Hantavirus-infected cells. Therefore, it could be concluded that Hantavirus-induced activation of IRF1 and IRF3 may lead to changes in cytokine and IFN inducible protein expression in infected cells.

DNA Array data have shown upregulation of several genes controlling processes of apoptosis, growth and proliferation. For example, upregulation of transcriptional activity of Bcl2 gene has been detected in Hantavirus infected HUVECs. Also, Hantavirus infected cells are characterized by transcriptional activation of vascular endothelial growth factor (VEGF), a survival factor for endothelial cells, which prevents apoptosis by inducing Bcl-2 expression. It has been shown that VEGF and Bcl2 cooperate to prevent apoptosis *in vitro*. For instance, increased expression of Bcl-2 was found in neuroblastoma cells treated with VEGF. Also, VEGF abrogates apoptosis induced by TNF-α-induced serum starvation (Pidgeon et al., 2001; Beierle et al., 2002). Therefore, it could be suggested that activation of Bcl2 and VEGF can explain absence of apoptosis in Hantavirus infected cells.

It has been suggested that cytokines play important role in pathogenesis of the vascular leakage in Hantavirus infected microvascular beds (Zaki et al., 1995). DNA Array data have shown an increased expression of a cluster of CC chemokine genes including RANTES (CCL5; Khaiboullina et al., 2004). Also, data presented by Geimonen et al. (2002) have demonstrated that transcriptional activation of CCL5 is characteristic for HTNV and PHV infection of endothelial cells. It is known that CCL5 plays a role in regulation of immune effectors migration to the site of infection (Schall et al., 1990). Interestingly, mononuclear leukocyte accumulation is a histological hallmark of Hantavirus infection (Zaki et al., 1995). One could suggest that increased traffic of immune effectors through the endothelial monolayer may lead to its damage and, thereby, making it more permeable (Schall et al., 1990).

## N Protein’S Role In Regulation Of Antiviral State

Expression of the glycoproteins of the pathogenic hantaviruses inhibits INF-β and TBK-1 induction via virulence determinants present on the G1 cytoplasmic tail. However, it has been suggested that, this alone may not be sufficient to make them virulent and some other virulence factors may play role (Matthys et al., 2011). Recently, it has been demonstrated that the ANDV N protein hinders autophosphorylation of TBK1 resulting in the inhibition of IRF3 phosphorylation and RIG-I/MDA5-directed type I IFN induction (Cimica et al., 2014). Additionally, the N protein can affect the protein kinase R (PKR) dimerization (Wang and Mir, 2015). It has been demonstrated that the Hantavirus N protein prevents PKR phosphorylation, which is essential for its enzymatic activity. PKR inhibits virus replication and is essential for establishing antiviral state (Goodbourn et al., 2000). PKR activates IFN via NF-κB and IRF1 up-regulation (Kumar et al., 1997). Additionally, PKR can activate apoptosis in infected cells (Gil and Esteban, 2000). Therefore, it could be suggested that the glycoproteins and the N protein may interfere with antiviral activity in infected cells, thus promoting viral replication.

## Summary

There are two clinical entities associated with Hantavirus infection, HFRS and HPS. The mortality rate may vary from 0.1 to 40% depending on the particular Hantavirus involved. The Hantavirus genome is composed of a three negative sense single stranded RNA segments coding for the N protein, G1 and G2 glycoproteins and viral polymerase. Genetic reassortment between different hantaviruses has been documented both in nature and *in vitro*. Such reassortment could result in emergence of the novel Hantavirus strains with new virulence characteristics and/or new host range.

Emerging evidence suggests that the Hantavirus N protein plays a major role not only in virus replication, transcription and virus assembly, but also in establishing favorable environment for virus replication within the host cell. Pathogenic hantaviruses cause more pronounced changes in transcriptional activity of various cellular genes as compared to non-pathogenic strains. Activation of CCL5 may contribute to Hantavirus-induced leukocyte accumulation in infected tissue and, potentially, to pathogenesis of vascular permeability. The Hantavirus N protein interacts with host proteins interfering with activation of the antiviral pathways in infected cells.

## Conflict of Interest Statement

The authors declare that the research was conducted in the absence of any commercial or financial relationships that could be construed as a potential conflict of interest.
